# Safety and Efficacy of Isolated Endoscopic Cyclophotocoagulation in Pseudophakic Patients with Primary Open-Angle Glaucoma—12-Month Follow-Up

**DOI:** 10.3390/jcm10184212

**Published:** 2021-09-17

**Authors:** Marta P. Wiącek, Tomasz Miszczuk, Andrzej Lipiński, Anna Machalińska

**Affiliations:** 1 First Department of Ophthalmology, Pomeranian Medical University, Powst. Wlkp. 72, 70-111 Szczecin, Poland; marta.p.wiacek@gmail.com (M.P.W.); mtmiszczuk@gmail.com (T.M.); 2 “Dom Lekarski” Medical Center, ul. Rydla 37, 70-783 Szczecin, Poland; andrzejlipinski45@gmail.com

**Keywords:** efficacy, endoscopic cyclophotocoagulation, glaucoma, glaucoma surgery, intraocular pressure, safety, side effect

## Abstract

Background: We evaluated the safety and efficacy of endoscopic cyclophotocoagulation (ECP) for eyes with primary open-angle glaucoma (POAG). Methods: We included a total of 104 pseudophakic eyes treated with ECP. Visual acuity and intraocular pressure (IOP, mmHg) measurements were evaluated preoperatively and on days 1 and 7 and 2 and 12 months postoperatively. IOP ≤ 15 or ≥30% reduction from baseline were defined as therapeutic success. Results: The mean baseline IOP was 23.89 ± 8.63, and it decreased significantly at the day 1 (16.25 ± 7.32, *p* < 0.0001), day 7 (17.81 ± 6.37, *p* < 0.0001), 2nd month (17.77 ± 8.54, *p* < 0.0001) and 12th month (16.42 ± 7.05, *p* < 0.0001) follow-up visits. Therapeutic success was achieved in 55 (61.80%) eyes at the 12-month follow-up. Patients with POAG duration longer than 10 years or those using alpha agonist eye drops had a lower rate of therapeutic success (odds ratio: 0.52, 95% CI = 0.32–0.85, *p* < 0.05 and odds ratio: 0.92, 95% CI = 0.55–0.95, *p* = 0.024, respectively). A longer disease course was associated with higher IOP values (Rs =+0.281; *p* = 0.024) postoperatively. The number of antiglaucoma medications decreased significantly from 2.55 ± 1.16 to 2.11 ± 1.14 (*p* = 0.003). The ECP complications included a minor IOP increase (9.37%), pupil irregularity (15.73%), and the presence of fibrin (3.29%). Conclusions: The ECP is an effective and safe option, especially in eyes with a shorter glaucoma course.

## 1. Introduction

Endoscopic cyclophotocoagulation (ECP) with clear corneal access or through the pars plana of the ciliary body is a modification of the well-established transscleral cyclophotocoagulation (TCP) procedure. The advantage of this approach over standard TCP and both micropulse and ultrasound cyclophotocoagulation (MCP) is the precise vision-guided destruction of a certain number of ciliary processes. The use of a minimal and suitably set power of the diode laser causes only minor damage to the adjacent tissues. Additionally, ECP can be performed as an isolated procedure regardless of lens status or combined with lens extraction. Interestingly, ECP does not constitute a contraindication to the choice of another surgical method. As a result, ECP procedures are gaining an increasing number of advocates [1,2].

Effective reduction of IOP in eyes after ECP treatment has been reported for almost all types of glaucoma, including primary open-angle glaucoma (POAG), primary angle-closure glaucoma (PACG), glaucoma secondary to neovascular or trauma, and congenital and normal tension glaucoma (NTG) [2,3]. Some authors [4] have reported a lower efficacy of ECP in pseudoexfoliative glaucoma (PEXG, 30.4%) and uveitic and paediatric glaucoma. A study comparing the efficacy of ECP in patients with glaucoma revealed a significantly greater reduction in IOP in patients with POAG than in subjects with PACG and PEXG [4].

Obviously, different types of glaucoma require personalized management. Moreover, the efficacy of treatment may vary depending on the course of the disease. In the available medical literature, studies describing the efficacy of ECP have often been conducted in heterogeneous groups of patients diagnosed with different types of glaucoma [3]. This may cause problems with understanding the indications for ECP or the prediction of IOP reduction following this procedure.

In addition, several definitions of therapeutic success and discrepancies in postoperative management, including various steroid regimens used by different authors, have led to inconsistencies in reports on the efficacy of ECP. Consequently, it is difficult to compare the outcomes of ECP among the reported studies [1,2,4,5,6,7]. This fact prompted us to perform a prospective interventional study to analyse the efficacy and safety of ECP therapy in a homogenous group of pseudophakic POAG eyes at a 12-month follow-up. Additionally, we aimed to identify factors influencing the efficacy of IOP reduction after ECP.

## 2. Materials and Methods

### 2.1. Subjects

A prospective interventional study of pseudophakic eyes with POAG treated with ECP was conducted. Consequently, enrolled patients underwent an isolated ECP surgery at “Dom Lekarski” Medical Center in Szczecin, Poland between July 2015 and September 2017. The surgical intervention was followed by a 12-month observation and data collection period. In total, 141 eyes (162 patients) were enrolled for the study. Ophthalmological examination directly before surgery, and then at days 1 and 7, and 2 and 12 months after ECP was performed. Patients who failed to attend more than 1 scheduled follow-up visit were excluded from the analysis (*n* = 37). As a result, in this study, 104 eyes with POAG from 90 Caucasian patients were considered for analysis.

The inclusion criteria for the study were as follows:

- POAG diagnosis,
- pseudophakic eye,
- a lack of satisfactory reduction in IOP with previous antiglaucoma treatment and/or progression of the disease based on the findings of static perimetry or optical coherence tomography of the optic disc.

The following conditions were defined as exclusion criteria:

- phakic and aphakic eyes,
- concomitant pseudoexfoliative syndrome or other type of secondary glaucoma,
- history of ocular surgery other than anti-glaucoma i.e., vitrectomy, corneal surgery,
- complicated cataract extraction or cataract surgery within past 3 months,
- previous ECP procedure,
- history of an eye trauma in the operated eye,
- iris neovascularization or proliferative retinopathy,
- history of chronic or recurrent uveitis,
- patients unable to give a written informed consent.

Patient demographic data on the duration of glaucoma, previous IOP-lowering surgical or laser procedures, antiglaucoma medication intake, and a family history of glaucoma were collected.

This study was approved by the local Bioethics Committee. Written informed consent for participation in the study was obtained from all patients.

### 2.2. Surgical and Postoperative Management

In all cases, ECP was performed according to the previously described surgical technique using a straight or curved 20 G endoscope (EndoOptiks, Inc., Beaver Visitech International, Little Silver, NJ, USA) [2]. The procedures were performed by two experienced operators (TM, *n* = 88; AL, *n* = 16). In all cases, cyclodestruction was performed through a clear corneal incision under topical proparacaine (Proxymetacaine) and intracameral 1% lidocaine local anaesthesia. Depending on the planned extent of laser therapy of the ciliary processes, ECP was performed either through a single clear corneal incision for the 270° range (*n* = 14) or two clear corneal incisions for the 360° range (*n* = 90). Subsequently, the ECP patients received the following topical medications: broad-spectrum fluoroquinolone antibiotic and nonsteroid anti-inflammatory drug were applied 4 times a day, as well as steroid drops were instilled in decreasing doses from 6 to 2 times a day for 3 weeks. Patients were recommended to continue to use their antiglaucoma medications, and their medication regimen was not modified within the first month after ECP.

### 2.3. Clinical Examination

The best corrected visual acuity (BCVA) and IOP were measured by the Snellen letter chart and Goldmann applanation tonometer [mmHg], respectively. The condition of the anterior segment of the eye was assessed in a slit lamp examination. All eyes were assessed carefully for the presence of any side effects of performed ECP. Accordingly, three major groups of ECP complications reported in previous studies and summarized by Kaplowitz et al. [8] were divided into: (i) corneal surface and anterior chamber defects; (ii) adverse effects of posterior segment; (iii) IOP disturbances. In the first group, the following conditions were evaluated: corneal oedema, Herpes simplex keratitis recurrence, hyphema, fibrin in the anterior chamber, iris inflammation and iris bombe. In the second group posterior segment of the eye was evaluated in order to assess vitreous haemorrhage, cystoid macular oedema, choroidal or retinal detachment. Additionally, IOP measurements were targeted to detect IOP spike, aqueous misdirection, persistent hypotony or phthisis. Consequently, indirect ophthalmoscopy was performed with a Volk 90 D lens to detect changes in the eye fundus that could result in complications after ECP.

An IOP value ≤ 15 mmHg or ≥30% reduction from baseline were defined as a therapeutic success, regardless of whether topical treatment was maintained. ECP was considered ineffective when the IOP was over 15 mmHg while maintaining topical treatment or a minimum 30% IOP reduction was not reached. Similarly, when additional topical medications or another surgical treatment were required, ECP failure was established. Additionally, the absolute decrease in IOP was defined as the mean reduction in IOP at consecutive follow-up examinations expressed in mmHg. A relative postoperative reduction in the IOP was established as a percentage of the baseline IOP value. In both analyses, only eyes with unchanged or decreased IOP were included.

### 2.4. Statistical Analysis

The normality of the data distribution was assessed using the Shapiro–Wilk test. The homogeneity of variance was evaluated with Levene’s test. A *t*-test was performed to assess the difference in age between the sex groups. In contrast, the Mann-Whitney U test was performed to evaluate changes in IOP values according to the operator or extension of the cyclodestruction. The Wilcoxon signed-rank test was used to evaluate changes in BCVA, IOP and number of medications as a result of ECP. Spearman’s rank correlation coefficient (Rs) test was used to assess the correlations between pre-ECP and post-ECP values of the IOP, as well as glaucoma duration. A univariate logistic regression analysis was performed to assess the effect of the selected individual variables on the reduction of the IOP ≤ 15 mm or 30% reduction from baseline. A statistical analysis was carried out using Statistica version 13.3 (TIBCO Software Inc., Palo Alto, CA, USA) software. Statistical significance was adopted at *p* < 0.05. The results are presented as the mean ± standard deviation unless marked otherwise for variables with a strong deviation from a normal distribution.

## 3. Results

### 3.1. Baseline Characteristics of the Study Group

The mean age of the study participants was 75.85 ± 7.87 years. In total 69 women and 36 men (mean age 75.35 ± 8.13 years vs. 76.78 ± 7.39 years, *p* = 0.438) were enrolled for the study. The analysis included 104 eyes at baseline, 83 eyes at 1 day, 91 eyes at 7 days, 64 eyes at 2 months and 89 eyes at the 12-month follow-up. Patients used at least one topical antiglaucoma medication in all surgically treated eyes. In some cases, laser treatment or another type of surgery was performed before ECP. The clinical characteristics of the study group are presented in Table 1.

### 3.2. Efficacy Evaluation

The mean baseline BCVA in the operated eye was 0.59 ± 0.34. No statistically significant differences in BCVA were detected at the seventh day visit (0.55 ± 0.32, *p* = 0.567), second month (0.62 + 0.32, *p* = 0.667) or 12th month visit (0.68 ± 0.31, *p* = 0.179) compared to preoperative values. At the 12-month follow-up, no case of BCVA reduction was noted.

**Figure 1:**
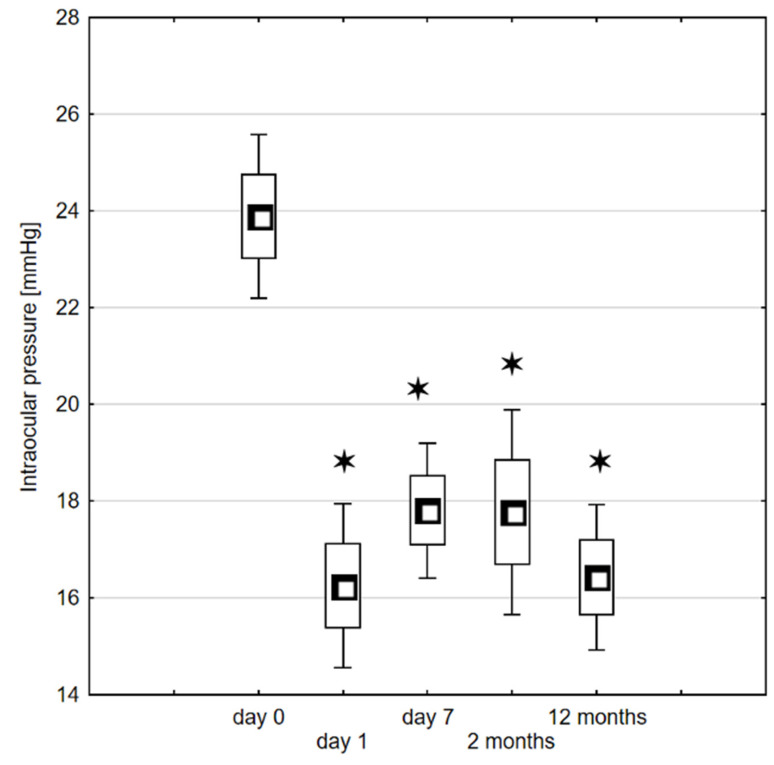
Boxplot representing the intraocular pressure (mmHg) measured on day 0, day 7 and at 2 and 12 months after endoscopic cyclophotocoagulation. The significance of the changes between the baseline and follow-up timepoints was assessed using the Wilcoxon signed-rank test. Level of significance: * *p* < 0.05.

The mean baseline IOP was 23.89 ± 8.63 (range 12.2–34.4) mm Hg. A significant decrease in IOP values was observed on the 1st day after ECP (16.25 ± 7.32 mmHg, *p* < 0.0001) and at the day 7 visit (17.81 ± 6.37 mmHg, *p* < 0.0001). Subsequently, a significant reduction in IOP was maintained at the 2nd month (17.77 ± 8.54 mmHg, *p* < 0.0001) and 12th month (16.42 ± 7.05 mmHg, *p* < 0.0001) postoperatively (Figure 1). As a result of ECP, therapeutic success was achieved in 54 eyes (65.06%) at the first day visit, 67 (73.63%) at the 7th day, 41 (64.06%) at the second month and 55 (61.80%) at the 12th month follow-up examination. Interestingly, in eyes that were classified as fulfilled a treatment success definition at 12th month follow-up, 2 (4.17%) did not obtain IOP values below 15 mmHg at day 1 visit. Accordingly, in three (6.25%) eyes at day 7 and in two (4.17%) eyes at second month follow up examination the IOP exceeded 15 mmHg. This might indicate that in selected cases, longer time period might be required to achieve satisfactory IOP reduction.

The calculated mean postoperative reductions in the IOP at consecutive time points are presented in Table 2. Moreover, the postoperative IOP values did not differ significantly between operators and did not depend on the extent of cyclodestruction (*p* > 0.05).

As a result of the ECP, a significant reduction in the number of antiglaucoma medications from 2.55 ± 1.16 to 2.11 ± 1.14 was found at the 12-month follow-up (*p* = 0.003). Consequently, the number of patients using two or more medications decreased significantly after ECP from 66 (63.46%) to 50 (56.17%). In six (6.74%) eyes, no topical antiglaucoma medications were used at 12 months post-ECP.

### 3.3. Impact of the Selected Parameters on Therapeutic Success

A univariate logistic regression analysis was performed to identify qualitative variables with an impact on the therapeutic success of ECP at the 12-month follow-up. The results of this analysis are presented in Table 3. Consequently, a lower rate of therapeutic success was observed in individuals with POAG duration longer than 10 years. Similarly, subjects with alpha agonist eye drop usage were also less likely to achieve therapeutic success. There was no influence of age, sex, type of previous glaucoma treatment, family history of glaucoma or extension of cyclodestruction on ECP success at the 12-month follow-up (Table 3). A history of TCP was not included in this analysis due to the low number of cases (*n* = 1). Interestingly, all eyes that achieved therapeutic success after ECP were naïve in terms of previous glaucoma surgery. This factor could not be included in the analysis.

A correlation analysis was performed to identify the influence of the pretreatment IOP values on the ECP efficacy. Consequently, a positive correlation between the baseline IOP and consecutive measurements during the follow-up was noted (Table 4). This means that eyes with higher pretreatment IOP values tended to have an increased IOP after ECP. Interestingly, a positive correlation between disease duration and the IOP values at the 12-month follow-up was observed (Rs = +0.281; *p* = 0.024). Moreover, the relative IOP reduction correlated negatively with IOP values at the 12-month follow-up visit (Rs = −0.263; *p* = 0.036). This indicates that patients with a longer duration of glaucoma had higher IOP values at the final follow-up examination. Additionally, in eyes with a longer disease course, the relative IOP reduction was decreased.

### 3.4. ECP Safety and Complications

No intraoperative complications of ECP occurred. However, in three eyes (3.29%), the presence of fibrin in the anterior chamber was found exclusively at 7 day post-operation. Additionally, in two cases minor corneal edema was observed on day 1 (2.41%) and day 7 (2.19%) after ECP but did not cause BCVA deterioration and resolved spontaneously with maintained standard eye drops regimen. Moreover, at the 12-month follow-up, pupil irregularity was diagnosed in 14 eyes (15.73%). No other complications in anterior segment were discerned. Although macular optical coherence tomography was not performed, no patients declared any vision deterioration with particular emphasis on central retinal function. Consequently, no vitreous haemorrhage, cystoid macular oedema, choroidal or retinal detachment were detected.

Contrarily, early postoperative complications included increased IOP over 21 mmHg in seven (8.43%) eyes at the 1-day visit and in 5 (5.49%) eyes at the 7-day visit. Subsequently, an IOP increase was noted in 6 (9.37%) eyes at the 2-month follow-up visit and in seven (7.86%) eyes at the 12-month follow-up visit. In comparison with the baseline values, an IOP increase of a mean value of 7.57 ± 11.49 mmHg was noted in eight (9.64%) eyes at the 1-day follow-up visit. Similarly, the IOP increased by 5.82 ± 10.06 in nine (9.89%) eyes at the 7-day visit, by 10.27 ± 7.61 mmHg in 6 (9.37%) eyes at the 2-month visit, and by 6.53 ± 7.97 mmHg in seven (7.86%) eyes at the 12-month visit. It is worth highlighting that no cases of aqueous misdirection, persistent hypotony or phthisis were observed. For three eyes (2.88%), further surgical treatment due to unsatisfactory reduction of the IOP was required. A trabeculectomy was performed in two eyes and TCP was performed for one eye. The mean time from ECP to reoperation was 3.83 ± 4.54 months. The follow-up of these patients ended at the moment of their qualification for another IOP-reducing surgical procedure.

## 4. Discussion

In this study, the 12-month postsurgical outcome in pseudophakic eyes with POAG after an isolated ECP procedure was evaluated. ECP provides unique selective vision-controlled destruction of the pigmentary part of the ciliary body processes responsible for aqueous humour production. Here, in the homogeneous group of eyes with POAG treated with the isolated ECP procedure, a significant IOP reduction was observed as early as day 1 after the surgery. Moreover, the IOP decrease remained stable during a 12-month follow-up period. Although evidence of the impact of isolated ECP on IOP is missing, several studies have described a substantial reduction in IOP as a result of an ECP procedure combined with cataract extraction. Consequently, Roberts et al. [6] noted an increased reduction in the IOP within a 6-month follow-up period. The average IOP decrease was 6–7.1 mmHg after combined ECP and phacoemulsification [3,9].

Importantly, the majority of papers published to date are retrospective studies based on eyes with various types of glaucoma. According to Morales et al. [4], eyes with POAG presented a higher percentage of a satisfactory IOP reduction after ECP in comparison with other types of glaucoma. In contrast, Francis et al. [10] described an initial decrease from the preoperative IOP value of 12 mmHg with a temporary increase of 10 mmHg at the 12- and 24-month follow-ups, followed by an average 13 mmHg reduction at the 36-month follow-up. Although the authors designed a prospective study exclusively in POAG eyes, the exact influence of ECP due to the inclusion of eyes with combined cataract surgery was not possible in this population. On the other hand, Chen et al. [11] retrospectively analysed the isolated ECP outcome in eyes with several types of refractory glaucoma and found that the mean IOP reduction was 10.7 mm Hg at the mean 12-month follow-up, which constituted a decrease of 34% from baseline IOP. However, that study population was not homogenous in terms of previous surgeries or lens status and applied the pars plana approach, which could introduce a confounder into the results. It is worth highlighting that to the best of our knowledge, this is the first prospective analysis that included exclusively pseudophakic eyes with POAG treated with ECP. This study design enabled a detailed evaluation of the procedure’s efficacy and dynamics of the IOP changes at a 12-month follow-up.

The clinical value of the IOP decrease might be expressed by a therapeutic success rate. Since the target IOP is expected to be established individually according to the severity of glaucomatous damage, several definitions of therapeutic success have been introduced in the literature [4,6,12,13,14]. Due to the inclusion of POAG eyes without particular emphasis on a selected disease severity in this study, we adopted a definition of IOP below 15 mmHg regardless of whether topical treatment was maintained as the most universal and safe method for the prevention of glaucomatous optic nerve damage and functional progression. As a result, in this study, at the 1-day examination, therapeutic success was achieved in 57.83% of eyes and maintained in 53.93% at the 12-month follow-up time. Similarly, Morales et al. [4] reported that therapeutic success set at 15 mmHg was achieved in 72.3% of eyes at an average follow-up of 17 months. Additionally, in 11.9% of eyes, a satisfactory IOP was maintained without topical antiglaucoma burden. The implementation of a combined ECP procedure with phacoemulsification in the study by Morales et al. [4] impeded the inference of an impact of isolated ECP surgery on the IOP and consequently led to a discrepancy between their and our results. Notably, some researchers defined therapeutic success at a level of 21 mmHg [12,14]. Since this is considered to be a normal IOP in healthy eyes, in the case of glaucoma, the target IOP should be set individually according to the degree of disease advancement and the patient’s life expectancy to prevent optic nerve damage. Interestingly, Chen et al. [11] noted that the percentage of eyes with an IOP lower than 21 mmHg decreased over time up to the 2-year follow-up.

Additionally, the inclusion of various patients with different disease durations in the homogenous POAG group enabled us to analyse the impact of both previous topical and surgical treatment on the ECP outcome. Interestingly, all eyes in the group that achieved therapeutic success were naive in terms of previous glaucoma surgeries. Our observation is consistent with Morales et al. [4], who reported a double risk of failure in eyes with a history of IOP-lowering procedures. However, in their retrospective study, a wide range of 80 to 360 degrees of cyclodestruction was performed for various types of glaucoma. Consequently, the lack of homogeneity in the study population along with discrepancies in surgical techniques makes it impossible to predict the impact of previous glaucoma treatments on the ECP outcome. In contrast, Roberts et al. [6] did not detect any significant differences in the ECP outcomes depending on previous surgical or laser treatment of glaucoma in retrospective analyses. The inclusion of various types of glaucoma followed by low 50% attendance at follow-up visits in their study should be considered significant confounders. It is worth mentioning that in several previous studies, ECP was reserved for eyes with glaucoma refractory to other treatment modalities, including various surgical methods [4,12,14,15]. On the other hand, several studies considered a history of antiglaucoma surgery prior to ECP as an exclusion criterion for further analysis [10,11,13]. Our findings indicate that ECP seems to be particularly beneficial for patients with shorter disease course and without a history of previous glaucoma surgery [1,2].

In addition, the univariate logistic regression analysis revealed factors that significantly increased the chance of obtaining therapeutic success after ECP at the 12-month follow-up. Consequently, a disease duration not exceeding 10 years promoted a chance of decreasing the baseline IOP to 15 mmHg. In contrast, we revealed that the usage of alpha agonists decreased the chance of achieving therapeutic success at the 12-month follow-up after ECP. Since alpha agonists are not considered a first-line treatment in glaucoma, this observation supports the hypothesis that eyes with a longer disease duration or more advanced glaucoma are less likely to obtain a satisfactory IOP as a result of ECP. To date, this is the first study that evaluated both the cumulative number of eye drops and monitored the impact of various groups of antiglaucoma drops on the ECP outcome.

Interestingly, previous retrospective studies revealed that a higher baseline IOP was correlated with a greater IOP reduction at the 12-month follow-up [6,15]. Consequently, we detected a more pronounced decrease in IOP in eyes with a higher IOP preoperatively.

As a result of the significant IOP reduction, the number of antiglaucoma agents could be decreased. According to the literature, ECP allows for the maintenance of individual target IOPs with a substantial reduction or cessation of the use of antiglaucoma eye drops [2,3,4,6]. Importantly, there is some evidence that ECP is associated with greater patient compliance [13]. The present study confirmed a significant reduction in the average number of antiglaucoma medications. Moreover, a reduction in the number of patients using two or more medications was noted. Similarly, Kang et al. [3] reported a mean 29.6% reduction in the number of drugs (from an average of 2.7 to 1.9). In a study by Morales et al. [4], 75% of patients used more than three antiglaucoma medications at baseline, and this number decreased significantly to 46% after ECP [4]. However, both reports were based on retrospective analysis of a combined ECP and phacoemulsification surgery performed in eyes with various glaucoma subtypes by several surgeons. Consequently, various inclusion criteria and management regimens may lead to some discrepancies between studies in the reduction of antiglaucoma agents. In our study, the influence of isolated ECP in eyes with POAG was not dependent on the concomitant phacoemulsification procedure, surgical extension or type of glaucoma.

Although ECP might be considered a minimally invasive method thanks to its conjunctival spearing approach, some complications of this procedure have been described [1,2,3,4,15,16,17]. The present study demonstrated increased early postoperative IOP, minor corneal edema, fibrinous exudate and pupil distortion as well as postsurgical complications. According to the literature, the IOP spike in the early postoperative period might be related to retained viscoelastic material in the anterior segment of the eye after ECP. Moreover, the local inflammatory process as a response to tissue destruction of the pigmentary processes may require more intensive steroid treatment [2,3,17]. Importantly, our observations are consistent with previous reports that full-range 360° ablation did not increase the incidence of severe complications such as hypotony, subluxation or displacement of the lens [3].

Despite of the fact that authors of this study put some effort to provide reliable results, some limitations might be pointed out. The analysed group was not homogenous in terms of the pre-ECP therapeutic management including the antiglaucoma surgeries or the glaucoma burden before the procedure. However, the inclusion criteria created an opportunity to analyse the factors that correlated with successful outcome of the procedure. In addition, ECP were done by two operators and in two extensions (270° and 360°). Nevertheless, according to the performed analysis, the postoperative IOP reduction depended neither on operator nor on ECP extension. Differences in the ECP efficacy according to glaucoma duration and its’ previous interventional treatment observed in this study inspire to further investigation of this phenomenon in POAG eyes.

## 5. Conclusions

In summary, the design of this study provided a unique opportunity to carefully follow patients long-term for changes in IOP dynamics after isolated ECP surgery. Our study evaluated the safety and efficacy of an isolated ECP procedure in a homogenous group of pseudophakic eyes with POAG at a 12-month follow-up. A significant reduction in IOP was observed as early as the first day after ECP and was maintained until the 12-month follow-up examination. Interestingly, a longer disease course was correlated with a lower IOP reduction. Moreover, patients with POAG durations longer than 10 years or those using alpha agonist eye drops had a lower rate of therapeutic success at the 12-month follow-up. As a result of ECP, a significant decrease in the use of antiglaucoma eye drops was noted. In summary, ECP is an efficient method of antiglaucoma treatment with a low risk of postoperative complications, especially in eyes with POAG naïve to previous glaucoma surgeries with a disease course of less than 10 years.

## Figures and Tables

**Table 1:** Clinical characteristics of the patients treated with endoscopic cyclophotocoagulation.

Analyzed Variables	
Duration of glaucoma [years]	8.65 ± 5.82
Number of antiglaucoma medications used before ECP	2.55 ± 1.16
Medications reducing the IOP	87 (83.65%) prostaglandin analogs
	76 (73.08%) beta blockers
	62 (59.61%) carbonic anhydrase inhibitors
	34 (32.69%) alpha agonists
IOP-lowering procedures before the ECP	34 (32.69%) ALT
	26 (25.0%) SLT
	15 (14.42%) trabeculectomy
	1 (0.96%) TCP
1st degree relatives with glaucoma	25 (24.04%)

Abbreviations: IOP, intraocular pressure; ECP, endoscopic cyclophothocoagulation; ALT, argon laser trabeculoplasty; SLT, selective laser trabeculoplasty; TCP, transscleral cyclophothocoagulation.

**Table 2:** Mean values of absolute and relative postoperative reduction of IOP depending on the time after endoscopic cyclophotocoagulation.

	Follow-Up Time Point			
	Day 1	Day 7	2 Months	12 Months
Percentage of the patients with decreased IOP after the ECP [%]	86.54	91.52	87.5	89.55
Mean absolute reduction of the IOP [mmHg]	8.17 ± 10.52	5.94 ± 9.95	5.49 ± 9.25	7.62 ± 8.05
Mean relative reduction of the IOP [%]	27.97 ± 39.47	18.83 ± 33.12	19.96 ± 32.37	28.03 ± 26.27

Abbreviations: IOP, intraocular pressure; ECP, endoscopic cyclophotocoagulation.

**Table 3:** Evaluation of the selected parameters influencing the therapeutical success of endoscopic cyclophotocoagulation by the univariate logistic regression method.

Analysed Parameter	Variants	Odds Ratio	95% CI	*p* Value
age [years]	≤75	0.93	0.58–1.48	0.762
	>75	1.07	0.67–1.71	
sex	female	1.53	0.95–2.46	0.081
	male	0.65	0.41–1.06	
glaucoma duration [years]	≤10	1.92	1.17–3.16	<0.001
	>10	0.52	0.32–0.85	
family history of glaucoma	yes	0.97	0.56–1.66	0.931
	no	1.03	0.6–1.76	
ECP extension	270°	0.85	0.48–2.03	0.549
	360°	1.19	0.54–2.54	
treatment prior to the ECP				
prostaglandin analogs	yes	1.38	0.77–2.48	0.279
	no	0.72	0.4–1.3	
beta blockers	yes	0.94	0.57–1.56	0.818
	no	1.06	0.64–1.75	
carbonic anhydrase inhibitors	yes	0.62	0.37–1.04	0.062
	no	1.6	0.96–2.66	
alpha agonists	yes	0.92	0.55–0.95	0.024
	no	1.08	0.64–0.82	
laser	yes	0.68	0.92–2.36	0.099
	no	1.48	0.42–1.08	
ALT	yes	0.44	0.26–0.74	0.203
	no	2.26	1.35–3.8	
SLT	yes	0.96	0.56–1.64	0.883
	no	1.04	0.6–1.78	
trabeculectomy	yes	0.61	0.32–1.16	0.133
	no	1.65	0.86–3.16	

Abbreviations: CI, confidence interval; ECP, endoscopic cyclophotocoagulation; ALT, argon laser trabeculoplasty; SLT, selective laser trabeculoplasty.

**Table 4:** Correlations between the baseline intraocular pressure and selected parameters in eyes after endoscopic cyclophotocoagulation.

Follow-Up Time Point	Day 1		Day 7		2 Months		12 Months	
	Rs	*p* Value	Rs	*p* Value	Rs	*p* Value	Rs	*p* Value
IOP [mmHg]	0.429	0.001	0.15	0.256	0.424	0.003	0.346	0.004
Mean absolute reduction of the IOP [mmHg]	0.561	<0.0001	0.686	<0.0001	0.65	<0.0001	0.721	<0.0001
Mean relative reduction of the IOP [%]	0.292	0.036	0.575	<0.0001	0.515	0.0001	0.513	<0.0001

Abbreviations: IOP, intraocular pressure; Rs, Spearman’s rank correlation coefficient. Spearman’s rank correlation coefficients between baseline IOP and various parameters at the corresponding time points.

## Data Availability

Data are available from the corresponding author on demand.
